# Supplementary material for: Volumetric finite-element modelling of biological growth
Source: Open Biol. 2019 May 29;9(5):190057. doi: 10.1098/rsob.190057 (PMC6544983; doi:10.1098/rsob.190057)
Supplement: Supplementary information [file rsob190057supp1.pdf]

Volumetric finite element modelling of biological growth: Supplementary information

Richard Kennaway* and Enrico Coen
Cell and Developmental Biology, John Innes Centre, Norwich, U.K.
*kennawar@nbi.ac.uk

Resources

The software described here is a further development of a Matlab package called GFtbox (short for Growth Factor Toolbox), and is available for download from Sourceforge at `https://sourceforge.net/projects/gftbox/`.

It can be difficult to convey three-dimensional data by static two-dimensional illustrations. We have therefore provided movies of some of the developments, and three-dimensional realisations of some of the illustrations in the supplemental files.

The 3D models are in VRML format (Virtual Reality Modelling Language), and can be viewed with a VRML viewer. The following viewers are available for current platforms and are free:

InstantReality. This will run on OSX 10.14 (Mojave), and Windows 7, 8, and 10. Downloadable from `http://www.instantreality.org`. This is a stand-alone application for viewing local files. (On Windows, it may complain of a missing file MSVCP110.DLL. This can be obtained from Microsoft by downloading and installing "Visual C++ Redistributable for Visual Studio 2012 Update 4". NB. 2012, not more recent versions. A web search is the best way to locate this on Microsoft's web site. Do not download the missing file from third-party sites.)

Cortona3D Viewer. Windows only. This installs as a plugin for Internet Explorer, and will view files either locally or over the web. It is downloadable from
`http://www.cortona3d.com/cortona3d-viewers`.

Methods

Our previous paper [1] includes a statement of the partial differential equations involved and an outline of the finite element method of numerical solution, to which we refer the reader for further information.

The running time for the models presented here, and for other models appearing in some of the cited publications, is generally of the order of seconds to tens of seconds per time step, on Macs and PCs typically several years old. The number of time steps for the models here is about 20 to 40, and as much as 200 for some other models. The running time for one step depends mainly on the number of finite elements, and in practice one uses as fine a subdivision and as short a time step as can be calculated within the time one wishes to allow. We have not explored techniques of employing the GPU to accelerate the calculations, and we have used cluster machines only to run multiple versions of a model simultaneously, rather than a single model faster.

As a check on the accuracy, we have tested the models with a mesh twice as fine (and therefore with 8 times as many elements). These take some hours to complete a single run, but give results scarcely

different from the lower-resolution versions.

Constitutive equations

Here are the equations governing elasticity, diffusion, and decay that are implemented by GFtbox.

Elasticity is linear, with the relation between stress σ and strain ϵ being [2]:

$$\sigma_{ij} \quad = \quad \sum_{kl} D_{ijkl}\, \epsilon_{kl} \tag{1}$$

The subscripts range over $1-3$, the spatial dimensions. D is the elasticity tensor, a fourth rank tensor with 81 elements, with certain symmetry properties implying that it has only 15 independent parameters. While GFtbox supports the use of arbitrary elasticity tensors, which may also vary over the material, the examples in this paper and in most of our use of GFtbox assume an isotropic and uniform elasticity tensor, for which the elasticity tensor simplifies to the form given in equation 2 below, with only two independent parameters. The equilibrium conformation is that which minimises the total deformation energy $\frac{1}{2}\sum_{ij}\sigma_{ij}\epsilon_{ij}$.

The transient diffusion-decay equation for a morphogen p is $\frac{dp}{dt} = K\nabla^2 p - Ap$, where K is its diffusion constant and A its decay rate. As with elasticity, GFtbox supports K and A that vary in time and across the tissue, but in all of the examples these are constant and uniform.

The steady-state diffusion equation is $\nabla^2 p = 0$.

Boundary conditions for the diffusion models take the form of fixing p to certain values at certain places.

GFtbox also supports setting rates of production of morphogens and interactions between morphogens.

Definition of the finite elements

The finite elements used in all the volumetric models presented here are first-order tetrahedra, with quadratic Gaussian integration. GFtbox also supports first-order pentahedra and hexahedra, also with quadratic Gaussian integration, but does not support remeshing for these.

Visualisation

For a solid object only the outside can be seen. Additional tools must be implemented to see inside, using cutaway sections or partial transparency. A field of polarisation arrows or tensor indicatrices on a surface is legible to the eye, but a cloud of such decorations in a spatial volume may be more difficult to interpret. Tools were developed for creating legible 2D representations of these 3D objects, and for exporting to 3D formats either for postprocessing in 3D graphics tools or for presenting directly in web pages or augmented/virtual reality applications.

Grid lines are embedded in the tissue and move with it, to illustrate the deformation arising through growth. (The grid is independent of the finite element decomposition, which is generally much finer.) Poisson's ratio is zero unless otherwise stated. (The behaviour of all the examples is qualitatively not much affected by the value.) Within each figure, images are drawn to the same scale, except when scale bars indicate otherwise.

Distance and time are measured in arbitrary units. Strain is a dimensionless quantity. Growth rate and strain rate have dimension 1/time. In the examples, the tissues generally have a radius or semi-diameter of 1 unit, the maximum growth rate is 1 per time unit, and the examples are run for a time suitable for making the resulting form clear. The visualisations of residual strain rate should be considered to be of

the ratio of residual strain rate to maximum growth rate. Since the latter is always 1 this is numerically the same, but is a dimensionless quantity with a physical meaning.

Description of models

Here we give a systematic summary, figure by figure, of the GFtbox models illustrated.

When growth is isotropic ($k_{par} = k_{par2} = k_{per}$) we write k for the common value of the growth rates.

The organisers referred to in the text as +ORG, −ORG, +ORG2, and −ORG2 are in the GFtbox models named `ID_SOURCE`, `ID_SINK`, `ID_SOURCE2`, and `ID_SINK2`. Each of these takes a value of either 1 or 0 at each vertex of the mesh. When one of these defined to be 1 "near" a certain point of the surface and 0 elsewhere, the neighbourhood in which it is 1 is defined as all points subtending an angle of no more than $15°$ with the given point, and within a distance of 0.3 of the surface.

Except for Figure 16, the coordinate frame in all models, as depicted, has the x axis from front left to rear right, the y axis from front right to rear left, and the z axis upwards. Figure 16 has the x axis from rear left to front right and the y axis from front left to rear right.

The centre of the initial state of every model is at the origin of coordinates.

Figure 6

The initial tissue is a sphere of unit radius.

The growth pattern is set up in the initial state and thereafter moves with the tissue.

Growth is isotropic everywhere.

(a) $k = 1$
(b) $k = r^2$
(c) $k = 1 - r^2$

Figure 7

The initial tissue is a thin disc of unit radius, with an initial small perturbation to its flatness.

The growth pattern is set up in the initial state and thereafter moves with the tissue.

Growth is isotropic everywhere.

(a) $k = 1$
(b) $k = r^2$
(c) $k = 1 - r^2$

Figure 8

The initial tissue is a cube of diameter 2.

The growth pattern is set up in the initial state and thereafter moves with the tissue.

Growth is isotropic everywhere.

Growth rate is a function of the distance r from the centre:

$k = 0$ for $r \leq 1$, $k = (r - 1)/(r\sqrt{3} - 1)$ for $r > 1$ (and is therefore 1 at the corners).

Figure 9

(a,b,e,f) The initial tissue is a cube of diameter 2.

The growth pattern is set up in the initial state and thereafter moves with the tissue.

Growth is isotropic everywhere.

$k = (x + 1)/2$, hence 0 on the left-hand face and 1 on the right-hand face.

(c,d,g,h) The initial tissue is a thin square parallel to the xz plane, of diameter 2. The growth pattern is defined in the same way as for the cube.

Figure 10

The initial tissue is a sphere of unit radius.

The growth pattern is set up in the initial state and thereafter moves with the tissue.

Growth is isotropic everywhere.

(a–c) $k = (z + 1)/2$.

(d–f) ID_SOURCE $= 1$ near the point $z = 1$, and 0 elsewhere.

POL is fixed at 1 where ID_SOURCE $= 1$, and has diffusion constant 0.1 and decay rate 0.5.

Figure 12

The initial tissue is a cube of diameter 2, whose centre is at the coordinate origin.

ID_SOURCE $= 1$ where $x = -1$ and 0 elsewhere.

ID_SINK $= 1$ where $x = 1$ and 0 elsewhere.

POL is fixed at 1 where ID_SOURCE $= 1$ and 0 where ID_SINK $= 1$. It diffuses by the steady state diffusion equation.

$k_{par} = 1$, $k_{par2} = k_{per} = 0$.

Figure 13

The initial tissue is a sphere of unit radius. These models differ only in how the polarity is organised. For all images in the third column, $k_{par} = 1$, $k_{par2} = 0$, $k_{per} = 0$. For all images in the fourth column, $k_{par} = 0$, $k_{par2} = 1$, $k_{per} = 1$.

(a) ID_SOURCE $= 1$ near the north pole and 0 elsewhere.

ID_SINK $= 1$ near the south pole and 0 elsewhere.

POL is fixed at 1 where ID_SOURCE $= 1$ and 0 where ID_SINK $= 1$. It diffuses by the steady state diffusion equation.

(b) ID_SOURCE $= 1$ near the north pole and 0 elsewhere.

POL is fixed at 1 where ID_SOURCE $= 1$, and has diffusion constant 0.1 and decay rate 0.5.

(c) ID_SOURCE $= 1$ at all vertices on the equatorial plane.

POL is fixed at 1 where ID_SOURCE $= 1$, and has diffusion constant 0.1 and decay rate 0.5.

(d) ID_SOURCE $= 1$ at all vertices on the diametrical planes perpendicular to the x axis and the y axis.

POL is fixed at 1 where ID_SOURCE $= 1$, and has diffusion constant 0.1 and decay rate 0.5.

Figure 15

The initial tissue is a cube of diameter 2, whose centre is at the coordinate origin.

ID_SOURCE $= 1$ where $x = 1$ and 0 elsewhere.

ID_SINK $= 1$ where $x = -1$ and 0 elsewhere.

ID_SOURCE2 $= 1$ where $y = 1$ and 0 elsewhere.

ID_SINK2 $= 1$ where $y = -1$ and 0 elsewhere.

POL is fixed at 1 where ID_SOURCE $= 1$ and 0 where ID_SINK $= 1$.

It diffuses by the steady state diffusion equation.

POL2 is similarly determined by ID_SOURCE and ID_SINK.

$k_{par} = 1$, $k_{par2} = 0.7$, $k_{per} = 0.1$.

Figure 16

The initial tissue is a sphere of unit radius.

ID_SOURCE $= 1$ at all vertices on the equatorial plane and 0 elsewhere.

POL is fixed at 1 where ID_SOURCE $= 1$, and has diffusion constant 0.1 and decay rate 0.5.

(e) $k_{par} = 0$, $k_{par2} = 1$, $k_{per} = 1$.

(f) $k_{par} = 0$, $k_{par2} = 1$, $k_{per} = 0$.

(g) $k_{par} = 0$, $k_{par2} = 1$, $k_{per} = 0.7$.

(h) $k_{par} = 0$, $k_{par2} = (y - 1)/2$, $k_{per} = 0.7$.

(j) The petiole region is defined by ID_PETIOLE, which is 1 where $y \leq 0.6$ and 0 elsewhere. $k_{par} = 0$, $k_{par2} = 1$, $k_{per} = 1 -$ ID_PETIOLE.

Complications with elisions

If a tetrahedron has any of the thin forms of Figure 1 (a-c), it would be desirable to merge the sets of vertices that lie very close to each other, eliminating this tetrahedron, some or all of its faces, and every other tetrahedron that shares any of those faces, as in Figure 1(d-f). However, in some circumstances, merging adjacent vertices in this manner can create an invalid mesh. This is because to be compatible with the finite element method, all internal edges of the tetrahedral mesh should belong to at least three tetrahedra, and this condition may be violated. For example,consider the three tetrahedra shown in the upper left of Figure 2 (a) and in exploded view (b). The internal edge shown in blue is shared by all three tetrahedra. If we elide the edge marked in red in Figure 2 (a), we obtain tetrahedra which have all of their vertices in common (c) and the blue edge is now shared by only two tetrahedra, which is illegitimate. Figure 2(d) shows the exploded view. Either the two remaining tetrahedra are both flat, or one has been everted. Neither case is compatible with the finite element method. Thus before carrying out elisions, there must be a check as to whether they would generate a legitimate mesh. Alternatively, the two coincident tetrahedra could be eliminated by a transformation similar to that of Figure 3(iii), but again, the validity of the transformation would have to be checked. Due to these complications, we have not yet implemented elision transformations.

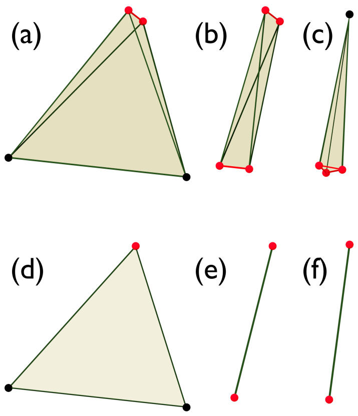

Figure 1: Desirable elisions for thin tetrahedra. (a–c) Three different thin tetrahedra with one (a), two (b) or three (c) edges (red) to be elided. (d–f) Results of eliding these edges.

There are also very thin tetrahedra that do not have any of their vertices close to each other. Four examples are shown in Figure 3 (a–d) which are all thin in the plane of the paper. In these cases flattening might be achieved by subdivision followed by elision. For example, in the tetrahedron shown in (a) a new vertex (red) could be introduced into the triangle on the back plane, allowing it to be subdivided into three. Eliding along the blue line would then lead to three flat triangles which may form tetrahedra with the surrounding mesh. Again the resulting mesh must be checked for validity (i.e. no two tetrahedra have the same vertices).

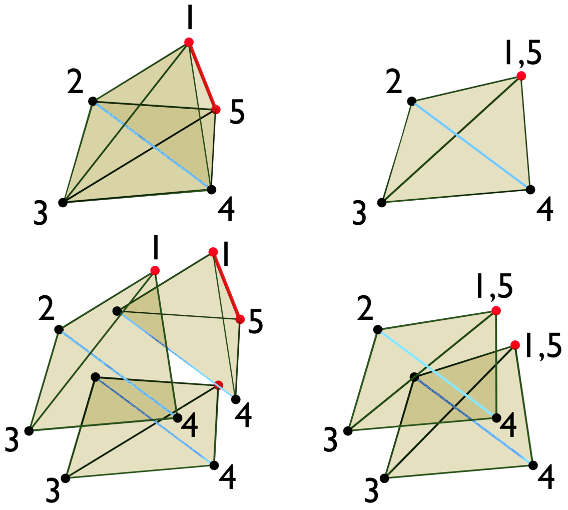

Figure 2: Elision of tetrahedra can yield an invalid mesh. (a) Mesh of three tetrahedra having a common edge (blue), where another edge (red) is to be elided. Vertices are numbered. (b) Exploded view. (c) The result of elision is an invalid mesh in which the two remaining tetrahedra have the same vertices. (d) Exploded view of the result.

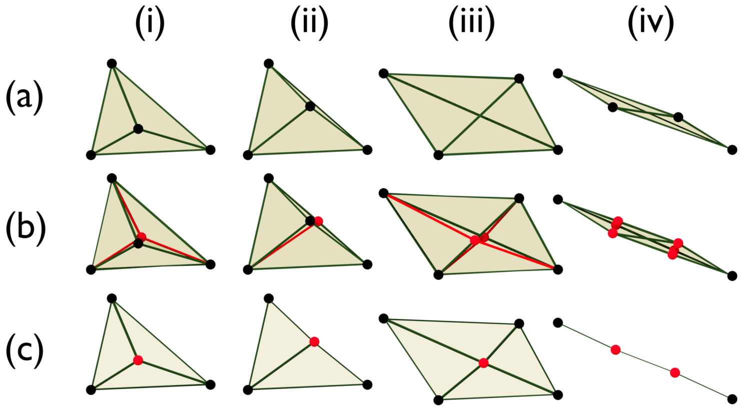

Figure 3: Flattening of thin tetrahedra through subdivision followed by elision. (a) Four types of thin tetrahedra with no short edges. These are all thin in the direction of view. In (i) a vertex is close to the interior of the opposite face. In (ii) a vertex is close to the interior of another edge. In (iii) two opposite edges pass very near each other. In (iv) the vertices are strung out in nearly a straight line. (b) Subdivision of each type of tetrahedron by introducing new vertices (red). This may require also subdividing tetrahedra that share edges with this one (not shown), to maintain consistency of the mesh. (c) Merging sets of vertices that are now close to each other eliminates the original tetrahedron. There are no tetrahedra in the resulting mesh fragments.

Mathematical derivations

Radially symmetric growth in a sphere

The symmetry of the spherical models of Figure 6 allows the equations of elasticity to be reduced to an ordinary differential equation that can be solved analytically. Comparing the result with that of the finite element calculation provides a validation test. We outline the calculation here, generalising the setting to a possibly hollow sphere in any number of spatial dimensions (i.e. including the case of a disc or annulus confined to the plane).

Suppose we have a possibly hollow sphere whose internal and external radii are $r_0 \geq 0$ and $r_1 > r_0$. Let it have a growth rate at each point of g_r in the radial direction and g_t in all tangential directions, each of these being a function only of r. We want to calculate the equilibrium configuration, assuming that it remains spherically symmetrical.

For generality, we solve the problem in an arbitrary number of dimensions $k+1$ (k being the number of independent tangential directions). The practical cases are $k=1$ and $k=2$. The first task is to determine the stiffness tensor of an isotropic elastic substance. The simplest expression of this tensor, valid in any number of dimensions, is in terms of Lamé's first and second parameters λ and μ (see e.g. [3], eqn. 5.4.2):

$$C_{pqrs} = \lambda\delta_{pq}\delta_{rs} + \mu(\delta_{pr}\delta_{qs} + \delta_{ps}\delta_{qr}) \qquad (p, q, r, s : 1 \ldots k+1) \tag{2}$$

C has three families of non-zero elements: $C_{pppp} = \lambda + 2\mu$, $C_{ppqq} = \lambda$ ($p \neq q$), and $C_{pqpq} = \mu$ ($p \neq q$).

By considering the effect of either an isotropic compression or a uniaxial compression we can express the Lamé parameters in terms of the bulk modulus K and Poisson's ratio ν:

$$\begin{aligned} \lambda &= \nu\frac{(k+1)K}{1+\nu} \\ \mu &= \frac{1}{2}(1-k\nu)\frac{(k+1)K}{1+\nu} \end{aligned}$$

μ is the shear modulus, which must be positive for a physically realistic material. Therefore $\nu < 1/k$. For convenience we define $\epsilon = 1 - k\nu$.

For a strain whose principal components are $\mathbf{v} = (v_1, ..., v_{k+1})$, the energy density, up to a constant factor is $\nu(\Sigma_i v_i)^2 + \epsilon\Sigma_i v_i^2$. (Notice that the bulk modulus K is part of that constant factor, as there are no external forces, only the material interacting with itself.)

Now let a spherically symmetrical deformation of the sphere be described by a function $\zeta(r)$ which maps the radius of a point in the original sphere to its radial displacement in the deformed sphere. Its new radius is thus $r + \zeta(r)$. The radial strain is $\rho = \zeta' - g_r$, and the tangential strain $\tau = \zeta/r - g_t$ (on each of k axes). Substituting these in the expression for energy density, multiplying by r^k (which is proportional to the area of the surface at radius r), and ignoring constant factors, we obtain the energy density per unit radius, which we write as:

$$L(r, \zeta, \zeta') = r^k\left(\nu(\rho + k\tau)^2 + \epsilon(\rho^2 + k\tau^2)\right)$$

The equilibrium conformation is obtained by minimising the integral of this from r_0 to r_1. By the calculus of variations, the integral is minimised if L satisfies the Euler-Lagrange equation:

$$\frac{\partial L}{\partial \zeta} - \frac{d}{dr}\frac{\partial L}{\partial \zeta'} = 0 \tag{3}$$

This works out to the basic equation:

$$r^2\zeta'' + kr\zeta' - k\zeta + r\alpha\epsilon(g_t - g_r) - r^2(g_r' + \alpha\nu g_t') \;=\; 0 \tag{4}$$

where $\alpha = \frac{k}{\epsilon+\nu}$.

At a free surface the boundary condition is:

$$\zeta' = g_r - \bar{\nu}(\zeta/r - g_t) \quad (5)$$

where $\bar{\nu} = \frac{1-\epsilon}{\nu+\epsilon}$. This is a quantity that we might call the dual Poisson's ratio, hence the notation, being the expansion in one direction resulting from a uniform compression in all the perpendicular directions. (For $k = 1$ this is identical to Poisson's ratio. For larger k it is larger.) For a solid sphere ($r_0 = 0$) the boundary condition at the centre is dictated by continuity: $\zeta(0) = 0$.

If g_t and g_r have the forms $a_t r^n$ and $a_r r^n$ for constants a_t and a_r, the equation can be solved analytically. The solution has the form:

$$\zeta = BP_n(r)r + Cr^{-k} + Dr \quad (6)$$

for constants B, C, and D, where $P_n(r) = \int_1^r r^{n-1}dr$. This is $(r^n - 1)/n$ for $n \neq 0$ and $\log r$ for $n = 0$. Defining the function P_n in this way avoids having to treat $n = 0$ as a special case. B is determined by substituting the general solution into Eq 4 (in which C and D cancel out), and C and D are obtained from the two boundary conditions.

Calculating these constants is complicated by several special cases that must be treated separately. In particular, if $\epsilon = 0$ the equation has no unique solution, as any volume-preserving flow can be added without violating the equations. In that case we can take the limit of the solution as ϵ tends to zero, which exists and has the same general form. The different boundary condition for $r = 0$ also implies a different calculation of the constants (C must be zero in that case). There is a further special case where $n = -k - 1$, for which the solution has a different form:

$$\zeta = B\log(r)r^{-k} + Cr^{-k} + Dr \quad (7)$$

the constants then being calculated in the same way.

Some special cases of the exact solution

Zero residual strain

For the radial and tangential growth rates to be such that there is no residual strain, we must have $\rho = \tau = 0$. This implies $g_r = g_t + rg_t'$. If $g_t = ar^n$, this implies $g_r = a(n+1)r^n = (n+1)g_t$. From $\tau = 0$ we obtain $\zeta = ar^{n+1}$ (which is the integral of g_r from 0 to r).

Uniform isotropic growth

Uniform isotropic growth is obtained when $n = 0$ and $a_r = a_t$. This implies that $C = 0$ and $D = a$, as we expect.

Isotropic non-uniform growth

The examples of Figure 6 have isotropic growth rate depending on radius of (a) 1, (b) r^2, and (c) $1 - r^2$. The initial sphere has radius 1 and Poisson's ratio zero. The exact solutions that are plotted in the figure are r, $0.4r + 0.2r^3$, and $0.6r - 0.2r^3$.

References

[1] Kennaway R, Coen E, Green A, Bangham A. Generation of Diverse Biological Forms through Combinatorial Interactions between Tissue Polarity and Growth. PLoS Comp Biol. 2011;7(6):22pp.

[2] Landau LD, Lifshitz EM. Theory of Elasticity (Course in Theoretical Physics, Vol.7). Butterworth-Heinemann; 1986.

[3] Slaughter WS. The Linearized Theory of Elasticity. Birkhäuser; 2002.
